# Effect of *Trichoderma harzianum* on maize rhizosphere microbiome and biocontrol of *Fusarium* Stalk rot

**DOI:** 10.1038/s41598-017-01680-w

**Published:** 2017-05-11

**Authors:** Kandasamy Saravanakumar, Yaqian Li, Chuanjin Yu, Qiang-qiang Wang, Meng Wang, Jianan Sun, Jin-xin Gao, Jie Chen

**Affiliations:** 1 School of Agriculture and Biology, Shanghai Jiao Tong University, Shanghai, P.R. China; 2 State Key Laboratory of Microbial Metabolism, Shanghai Jiao Tong University, Shanghai, P.R. China; 3 Key Laboratory of Urban Agriculture (South), Ministry of Agriculture, Shanghai, P.R. China

## Abstract

*Fusarium* stalk rot (FSR) caused by *Fusarium graminearum* (FG) significantly affects the productivity of maize grain crops. Application of agrochemicals to control the disease is harmful to environment. In this regard, use of biocontrol agent (BCA) is an alternative to agrochemicals. Although *Trichoderma* species are known as BCA, the selection of host-pathogen specific *Trichoderma* is essential for the successful field application. Hence, we screened a total of 100 *Trichoderma* isolates against FG, selected *Trichoderma harzianum* (CCTCC-RW0024) for greenhouse experiments and studied its effect on changes of maize rhizosphere microbiome and biocontrol of FSR. The strain CCTCC-RW0024 displayed high antagonistic activity (96.30%), disease reduction (86.66%), biocontrol-related enzyme and gene expression. The root colonization of the strain was confirmed by eGFP tagging and qRT-PCR analysis. Pyrosequencing revealed that exogenous inoculation of the strain in maize rhizosphere increased the plant growth promoting acidobacteria (18.4%), decreased 66% of FG, and also increased the plant growth. In addition, metabolites of this strain could interact with pathogenicity related transcriptional cofactor FgSWi6, thereby contributing to its inhibition. It is concluded that *T. harzianum* strain CCTCC-RW0024 is a potential BCA against FSR.

## Introduction

Maize (*Zea mays*) is an important grain crop for human food security, fodder, and biofuel production. But, its annual yield is significantly decreased due to the plant disease of *Fusarium* stalk rot (FSR) caused by the *Fusarium graminearum* (FG)^1, 2^. To overcome obnoxious effect of the plant disease, the farmers use several agrochemical pesticides. The input of the agrochemicals to the environment is proved to be harmful to beneficial microbiomes and considerably change the human attitude^3^. In this regard, it is obligatory to search for an alternative eco-friendly microbial source to prevent the plant disease. *Trichoderma* spp., are globally known as BCAs and used to prevent plant pathogens and increase plant immunity in field and greenhouse conditions^4^. This is because of their ability in beneficial interactions with plants (maize, cotton, cucumber) through production of auxin like compounds and secondary metabolites^5–7^. Therefore, the more studies on molecular mechanism of *Trichoderma* spp., and plants/microbiomes interactions are necessary^8, 9^. Moreover, the host specificity of microbial strain is an important concern in BCA application^10^, and selection of host-pathogen specific *Trichoderma* sp. is essential to increase its activity^11^. Bio-control activity of *Trichoderma* spp. is extraordinary due to multiple functions of the fungi such as mycoparasitism, antibiosis, and nutrient competition and production of enzymes, metabolites^4, 11–15^. Secondary metabolites (SMs) such as polyketides and peptaibols derived from the *Trichoderma* sp., have various biomedical and agriculture applications^16^. *Trichoderma* derived volatile organic compounds are reportedly promoting plant growth^17, 18^. Also *Trichoderma* are known to interact with the pathogens and inactivate the pathogenicity related proteins^19^. *Trichoderma* elicitor is also involved in the host-plant pathogen interaction^8, 9^. The protein elicitor *TVHYDII2* in the *Trichoderma viride* is reported to involve in establishment of plant root colonization and increase of the antagonist activity against pathogen^20^. In addition, *Trichoderma* spp. interacts with plant roots and induces systemic resistance against the pathogen^21^.

Association of plant and microbiome is crucial for plant health. Rhizosphere is an important interface involved in exchange of resources between plants and their soil environment^22^ because the plants communicate with their rhizosphere microbiome to control the pathogens^23^. Frequent input of the agrochemicals can reduce the phytopathogens, but it will also have a negative impact to plant associated microbiomes. In fact, beneficial microbial communities play significant role in the soil quality and fertility thereby maintaining the quality of native soil to ensure improved natural agriculture crop products. Hence the use of pathogen-specific microbial BCAs has advantage to specifically prevent the particular pathogen and to increase the beneficial rhizosphere microbiome and plant immunity.

BCAs of *Trichoderma* spp. have ultimate functions in promoting the plant beneficial microbial community and decreasing the pathogen attack through the specific interactions with host-pathogen. However, the effect of *Trichoderma* on changes of maize rhizosphere microbiome is poorly understood. According to the earlier report the transcriptional cofactor FgSWi6 is required for virulence and pathogenicity of FG^24^. Therefore, we hypothesize that inhibition of FgSWi6 can reduce the pathogenicity of FG. Hence, we screened a total of 100 *Trichoderma* isolates against FG by *in vitro* antagonistic assay. Based on screening experiments, we selected *Trichoderma harzianum* (CCTCC-RW0024) for greenhouse experiments to study its effect on changes of maize rhizosphere microbiome and biocontrol efficiency. The synergistic interaction between secondary metabolites and hydrolytic enzymes are crucial for BCAs^19, 25, 26^. Hence, we analyzed the biocontrol-related-hydrolytic enzymes, gene expression, and metabolites in the strain CCTCC-RW0024. In addition, we applied a molecular docking method to predict the inhibitory effect of *T. harzianum* metabolites on pathogenicity related transcriptional cofactor FgSWi6 of FG.

## Results

### Screening of biocontrol *Trichoderma*

In order to select the potent antagonist *Trichoderma* against FG, a total of 100 *Trichoderma* isolates were subjected to *in vitro* antagonist activity. Among the 100 isolates, the top ten potent isolates were selected for further experiments with high antagonist activity against FG such as *T. asperellum* strain CCTCC-SBW0102 (92.3%), *T. aureoviride* strain CCTCC-SBW0005 (85.2%), *T. harzianum* strain CCTCC-SBW0101 (89.2%), *T. asperellum* strain CCTCC-SBW0109 (93.4%), *T. asperellum* strain CCTCC-SBW0013 (77.4), *T. asperellum* strain CCTCC-SBW0052 (92.1%), *T. asperellum* strain CCTCC-SBW0091 (82.50%), *T. tawa* strain CCTCC-RW0023 (90.20%), *T. harzianum* strain CCTCC-RW0024 (96.3%) and *T. harzianum* strain CCTCC-SBW0181 (82.35%) (Supplementary Table 1).

### Biocontrol-related hydrolytic enzyme and gene expressions

The selected potent antagonist isolates were subjected to hydrolytic activity. The results are shown in Supplementary Table 2. The hydrolytic activity of chitinase (chitin; 85.7 ± 1.56%) protease (gelatin; 88.0 ± 1.58%) and glucanase (pachyman; 83.3 ± 2.54) was maximum in the strain CCTCC-RW0024 and minimum in strain CCTCC-SBW0005 (6.3 ± 1.56%). In case of cellulase the hydrolytic activity was maximum in the strain CCTCC-RW0023 (89.7 ± 4.56%).

Further, the relation between hydrolytic activity and antagonist activity of *Trichoderma* was analyzed. The results of correlation analysis revealed that the CWDEs such as chitinase, β (1–3) glucanase, protease and cellulase displayed a positive correlation with antagonistic activity. Thus the hydrolytic activity of chitinase and β (1–3) glucanase from *Trichoderma* could significantly increased the mycoparasitism of 19%, and 50% respectively (Supplementary Fig. 1).

**Figure 1:**
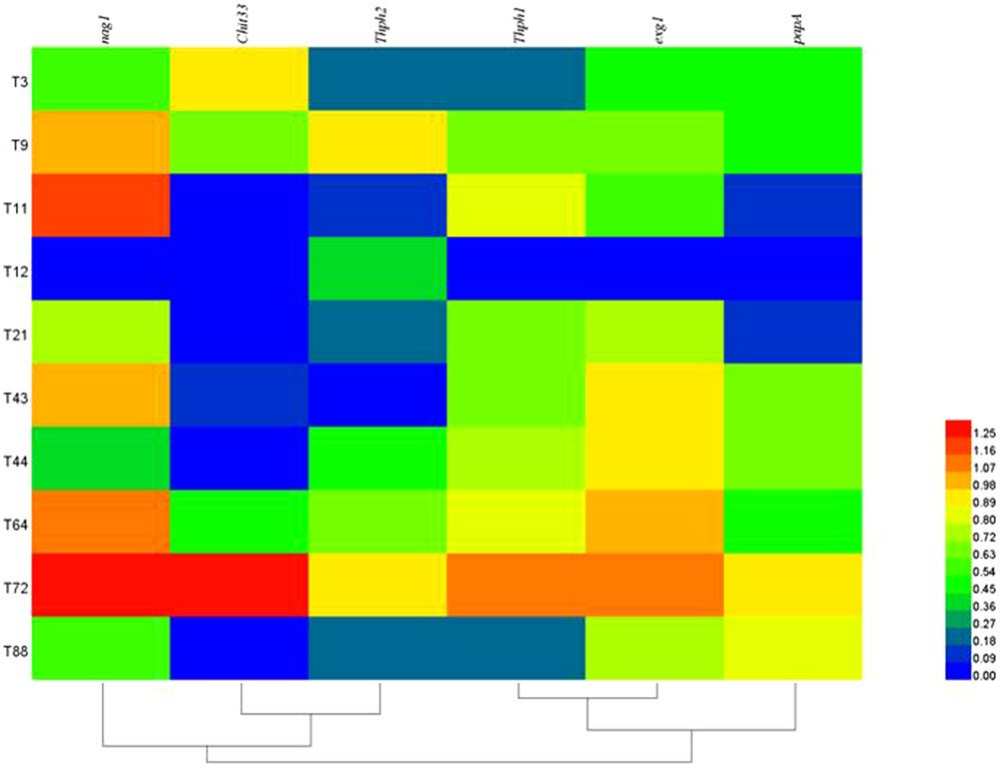
Heat map and clustering categorization of mycoparasitism related genes expression. Chitinase (*nag1, Chit33*), Cellulase (*Thph1* and *Thph2*), 1,3-β-Exoglucanase (*exg1*), Aspartyl protease (*papA*).

In this assay, we analyzed the expression of biocontrol-related gene expression in selected potent *Trichoderma* isolates. The results indicated that biocontrol-related gene expression of chitinase (*nag1, Chit33*), cellulase (*Thph1* and *Thph2*), 1,3-β-exoglucanase (*exg1*), aspartyl protease (*papA*) significantly varied between *Trichoderma* isolates. Among the tested isolates, the strain CCTCC-RW0024 were showed high mRNA related gene expression of biocontrol-related genes (Fig. 1).

### Effect of *Trichoderma harzianum* inoculation on physiological indices

**Figure 2:**
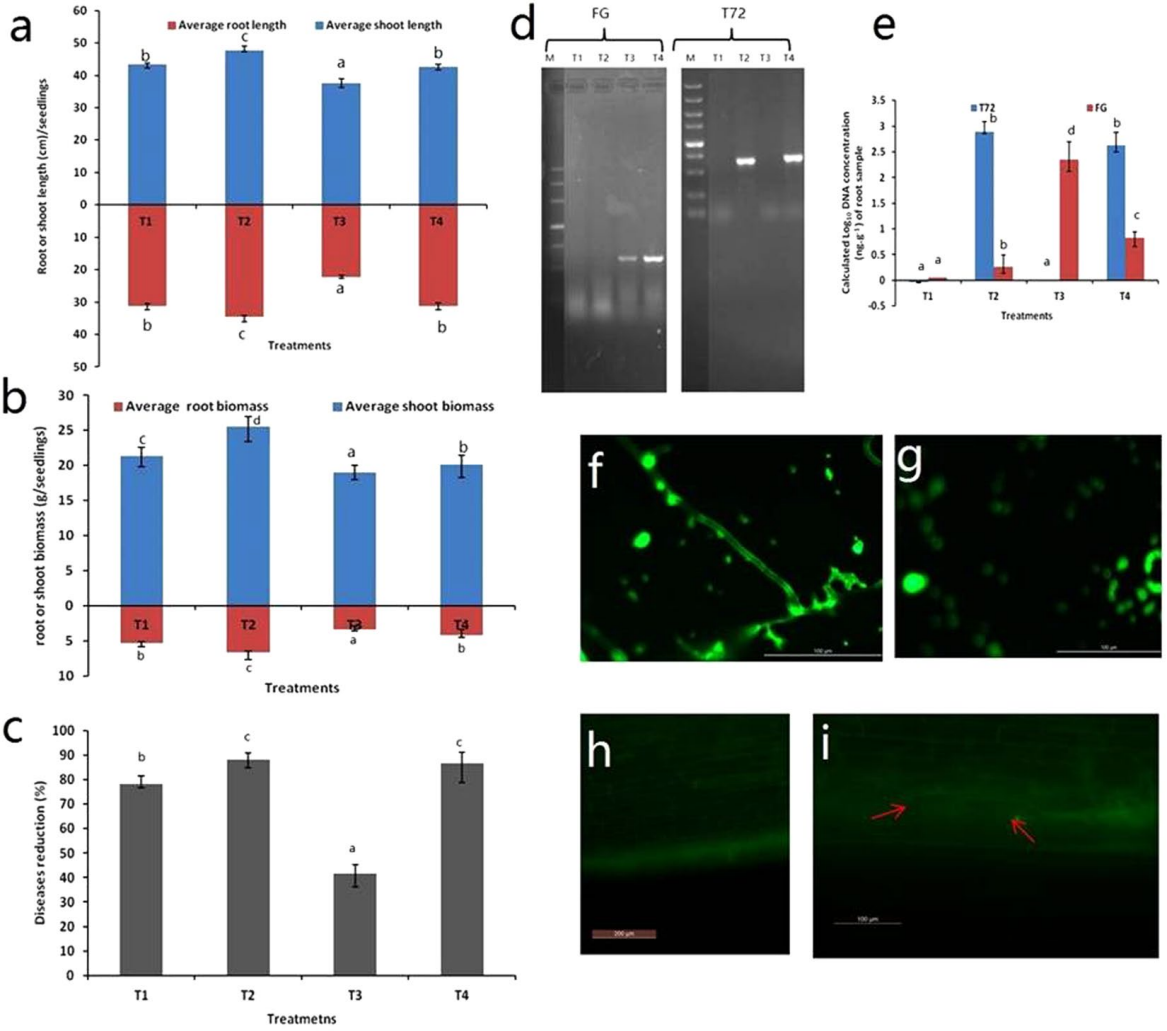
Analysis of physicological indices and root colonization of Trichoderma in maize root by qPCR and eGFP tagged *T. harzianum* strain CCTCC-RW0024. (**a**) Average root and shoot length (cm/seedlings), (**b**) root and shoot biomass (g/seedlings), (**c**) disease reduction (%), (**d**) electrophoresis representation of *T. harzianum* strain CCTCC-RW0024 and FG DNA presence in maize root DNA, (**e**) quantification of *T. harzianum* strain CCTCC-RW0024 and FG DNA in maize root by qRT-PCR, (**f**,**g**) confirmation of eGFP tagged *T. harzianum* strain CCTCC-RW0024 by microscopic observation of mycelium and spores respectively. (**h**) Uninoculated maize root control, (**i**) Observation of eGFP tagged *T. harzianum* strain CCTCC-RW0024 colonization in maize root highlighted in red arrow. (T1 (uninoculated), T2 (*T. harzianum* strain CCTCC-RW0024 alone), T3 (FG alone), T4 (Inoculated *T. harzianum* strain CCTCC-RW0024 and FG). T72- *T. harzianum* strain CCTCC-RW0024, FG- *Fusarium graminearum*. Results shown in bar diagram are mean ± S.E.M (n = 3), bars with the same letter are not statistically significant between the treatments following Duncan’s test (*p* < 0.05).

This experiment analysed the effect of exogenous application of *Trichoderma* or FG treatments on physiological changes in maize seedlings. Physiological indices were measured after one month of treatment in greenhouse experiment. The average shoot length (13.31%, *p* < 0.05), average root length (39.59%, *p* < 0.05), average shoot biomass (6.38%, *p* < 0.05), and average root biomass (23.52%, *p* < 0.05) increased significantly in T4 (*T. harzianum* strain CCTCC-RW0024 and FG inoculated) compared to T3 FG alone treated (Fig. 2a,b). The disease reduction varied significantly between the treatments and it was high in T4 (*T. harzianum* strain CCTCC-RW0024 and FG inoculated) and low in T3 FG alone treated (Fig. 2c). Thus, T4 showed that *T. harzianum* strain CCTCC-RW0024 significantly reduced the infection of FG, as evident by 86.66% diseases reduction and increased plant growth.

### Quantitative analysis of *T. harzianum* strain CCTCC-RW0024 in maize roots

This experiment studied the colonization of *T. harzianum* strain CCTCC-RW0024 in maize roots, by following two methods: (1) qRT-PCR (2) using eGFP tagged mutant of the strain by ATMT method. In the qRT-PCR method, we first designed the specific primers and confirmed the specificity by electrophoresis analysis. The results indicated that there was no visible band observed in uninoculated FG and/or strain CCTCC-RW0024 (Fig. 2d). Thus, designed primers could be accepted and used for the quantification of respective strains DNA in maize root. The calculated DNA of FG and strain CCTCC-RW0024 from qRT-PCR (Fig. 2e) showed that the DNA of strain CCTCC-RW0024 was found high in T2 (strain CCTCC-RW0024 alone treated; 2.88 ± 0.2 Log_10_ DNA concentration (ng.g^−1^) of root sample) and T4 (strain CCTCC-RW0024 and FG-treated; 2.62 ± 0.26 Log_10_ DNA concentration (ng.g^−1^) of root sample) whereas the FG was high in negative control T3 (FG alone treated; 2.35 ± 0.35 Log_10_ DNA concentration (ng.g^−1^) of root sample) and it significantly decreased in T4 (strain CCTCC-RW0024 and FG-treated; 0.82 ± 0.12 Log_10_ DNA concentration (ng.g^−1^) of root sample). In addition, the eGFP tagged strain CCTCC-RW0024 was confirmed root colonization by the observation of mycelium (Fig. 2f) and spores (Fig. 2g) under the fluorescent microscope. The microscopic observation and comparison of maize root treated and untreated eGFP tagged strain CCTCC-RW0024 clearly showed the successful colonization of the strain in maize root (Fig. 2h,i). This indicated that the strain CCTCC-RW0024 could significantly reduce the colonization of FG and its infections in maize seedlings.

### Exogenous application of *T. harzianum* on the maize rhizosphere microbiomes

**Figure 3:**
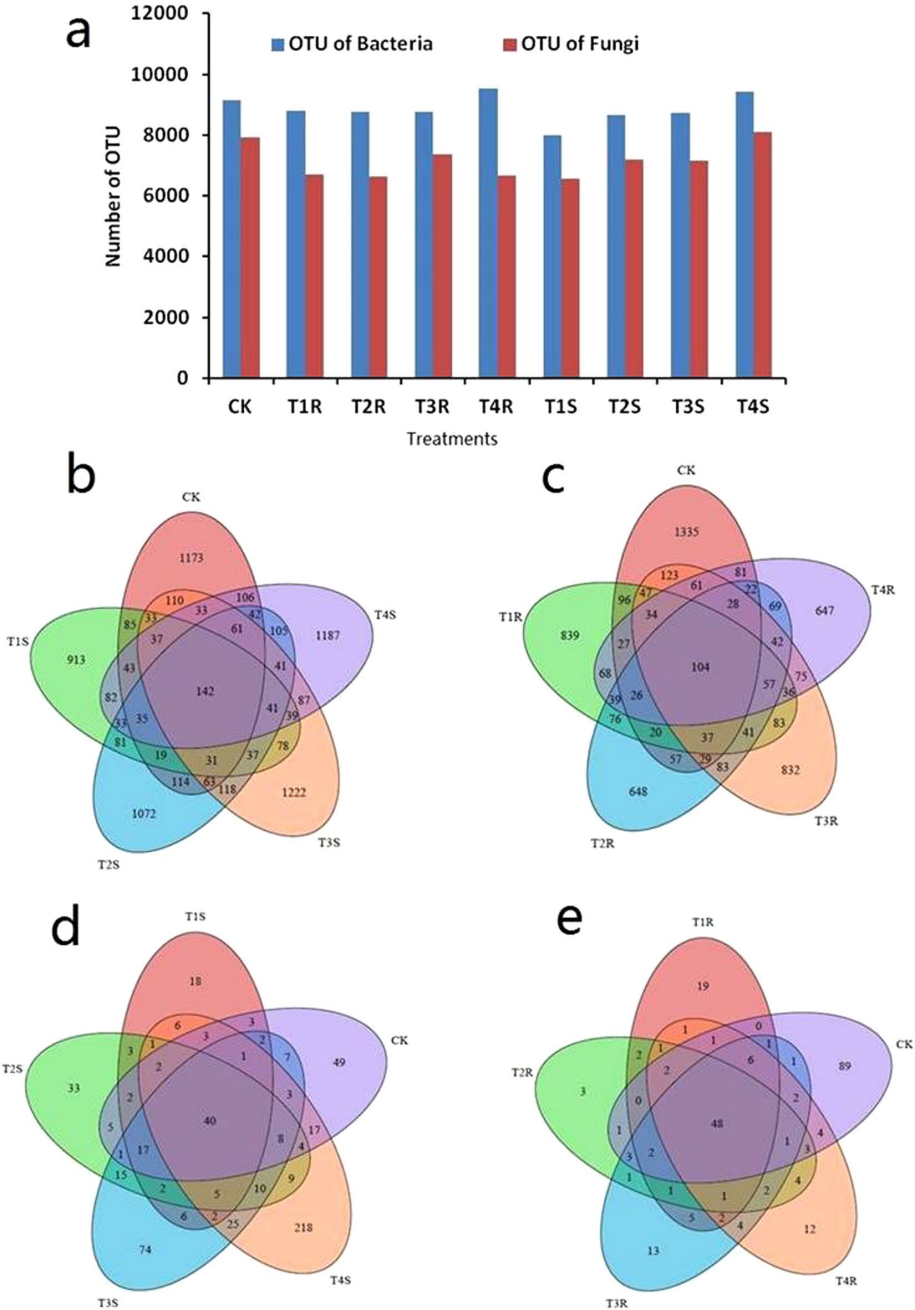
The distribution of operational taxanomical units (OTUs) found in maize root and rhizosphere soil with different treatment of Trichoderma and FG. (CK-unsterilized natural agriculture field soil,T1S- uninoculated soil, T2S-Soil inoculated with *T. harzianum* strain CCTCC-RW0024, T3S-Soli inoculated with FG, T4S-Soil inoculated with *T. harzianum* strain CCTCC-RW0024 and FG, T1R Maize root samples collected from uninoculated soil pot, T2R maize root collected from soil inoculated with *T. harzianum* strain CCTCC-RW0024, T3R- Maize root samples collected from inoculated with FG pot, T4R- Maize root samples collected from inoculated with *T. harzianum* strain CCTCC-RW0024 and FG).

In this experiment, we analyzed the exogenous application of *T. harzianum* on changes of maize rhizosphere microbiomes by pyrosequencing method. The sample collections and treatments are described in materials and methods. A total of 79812 bacterial OTUs and 64304 of fungal OTUs were subjected to the classification analysis. The dominant length distributions were 693 bp for fungi and 516 bp for bacteria. The OTUs of bacteria and fungi varied significantly between the treatments. The bacterial OTUs were found higher than the fungal OTUs recorded. However, the bacterial OTUs were the maximum of 9545 OTUs in T4R (strain CCTCC-RW0024 and FG-treated) and the minimum of 7987 OTUs in T1S (uninoculated), whereas the fungi OTUs was high in T4S (8085 OTUs) and low in T2R (6647 OTUs) (Fig. 3a).

The maize rhizosphere microbiome analysis between the treatments using the best-hit revealing the distribution and sharing of the same OTUs of bacteria in root and rhizosphere soil are shown in Fig. 3b,c. A total of 142 OTUs in rhizosphere soil and 104 OTUs in maize root were found common in different treatments. The CK (uninoculated) shared the maximum of 118 OTUs with T3S (FG alone treated) followed by 106 OTUs with T4S (strain CCTCC-RW0024 and FG-treated). In the case of fungi OTUs, 40 OTUs in rhizosphere soil and 48 OTUs in maize root were common with the treatments (Fig. 3d,e).

**Figure 4:**
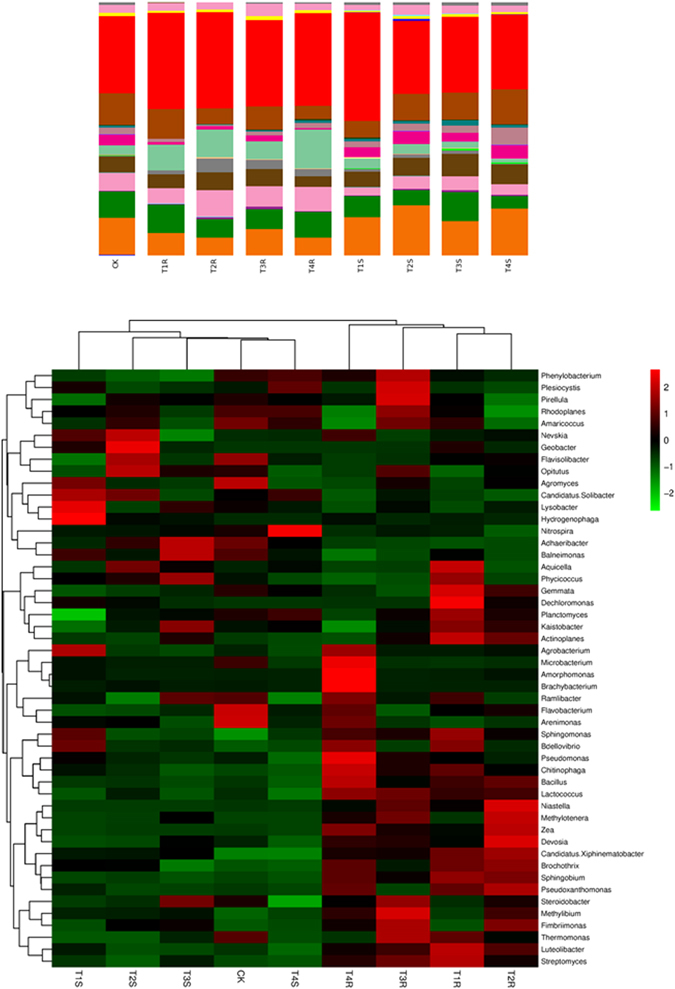
Analysis of bacterial OTUs (**a**) Taxonomical distribution of bacterial OTUs, (**b**) Heatmap analysis of OTUs of bacterial species based on the Bray- Curtis similarity of 454 pyrosequencing.

Taxonomical distribution of bacterial OTUs indicated that the acidobacteria followed by actinobacteria were dominant in the maize rhizosphere (Fig. 4a). The taxonomical distribution of the bacterial OTUs is shown in Supplementary Table 3. The results indicated that the acidobacteria were found higher in percentage in T2S (rhizosphere soil, strain CCTCC-RW0024 alone inoculated; 19.6%) and T4S (rhizosphere soil, strain CCTCC-RW0024 and FG inoculated; 18.4%) than that in T2R (rhizosphere root, strain CCTCC-RW0024 alone inoculated; 7%) and T4R (rhizosphere root, strain CCTCC-RW0024 and FG inoculated; 7%), whereas the actinobacteria were found higher in T3S (rhizosphere soil, FG alone inoculated; 11.6%) and T1R (rhizosphere root, uninoculated; 11.2%) and lower in T4S (rhizosphere soil, strain CCTCC-RW0024 and FG inoculated; 5%). In addition, the heat map showed the specific bacterial species distribution (Fig. 4b) and it indicated that the bacterial species counts were found higher in T4R (strain CCTCC-RW0024 and FG inoculated).

**Figure 5:**
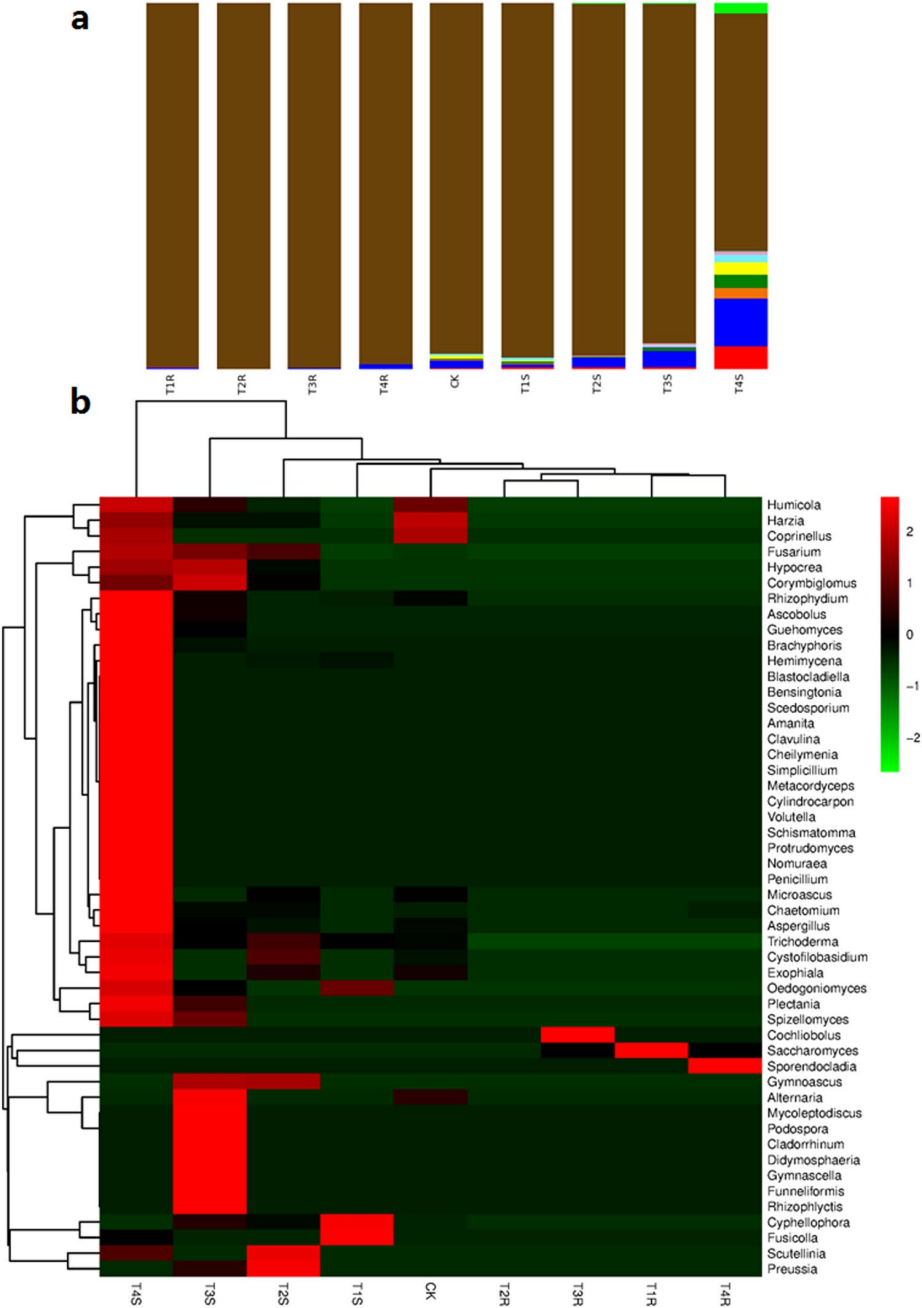
Analysis of fungal OTUs (**a**) Taxonomical distribution of fungal OTUs, (**b**) Heatmap analysis of OTUs of fungal species based on the Bray- Curtis similarity of 454 pyrosequencing.

Taxonomical distribution of fungal OTUs indicated that fungal distribution in maize root did not show significance with rhizosphere soil (Supplementary Table 4 and Fig. 5a); however, it was found similar to the treatments of T3R (rhizosphere root, FG alone inoculated) and T4R (rhizosphere root, strain CCTCC-RW0024, and FG inoculated). The Ascomycota followed by Basidiomycota were found higher in rhizosphere soil than that in the root. The Ascomycota was found maximum in T4S (rhizosphere soil, strain CCTCC-RW0024 and FG inoculated; 12.8%) and minimum in T4R (rhizosphere root, strain CCTCC-RW0024, and FG inoculated; 1.3%) and T3R (rhizosphere root, FG alone inoculated; 0.3%). The heat map analysis revealed that the fungal species was recorded higher in T4S (rhizosphere soil, strain CCTCC-RW0024 and FG inoculated). Interestingly *Trichoderma* sp., was found higher in T4S (rhizosphere soil, strain CCTCC-RW0024, and FG inoculated) and T2S (rhizosphere soil, strain CCTCC-RW0024 alone inoculated); whereas *Fusarium* sp., was found in the T2S (rhizosphere soil, strain CCTCC-RW0024 alone inoculated), T3S (rhizosphere soil, FG alone inoculated) and T4S (rhizosphere soil, strain CCTCC-RW0024 and FG inoculated; Fig. 5b).

### Extraction of fungal secondary metabolites

**Figure 6:**
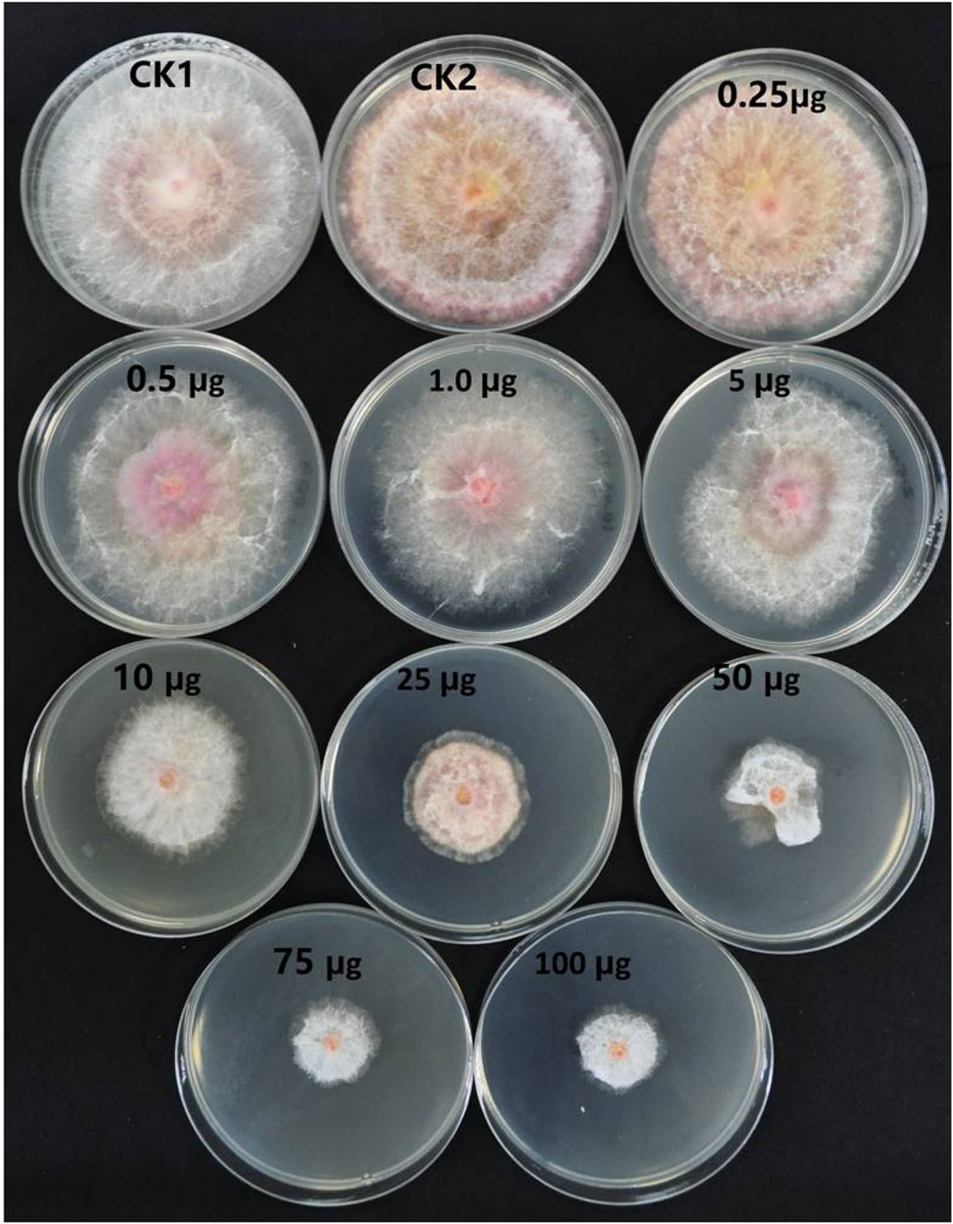
Effect of bioactive fungal metabolite derived from *T. harzianum* strain CCTCC-RW0024 (μl/100 ml of PDA) on growth of FG.

**Figure 7:**
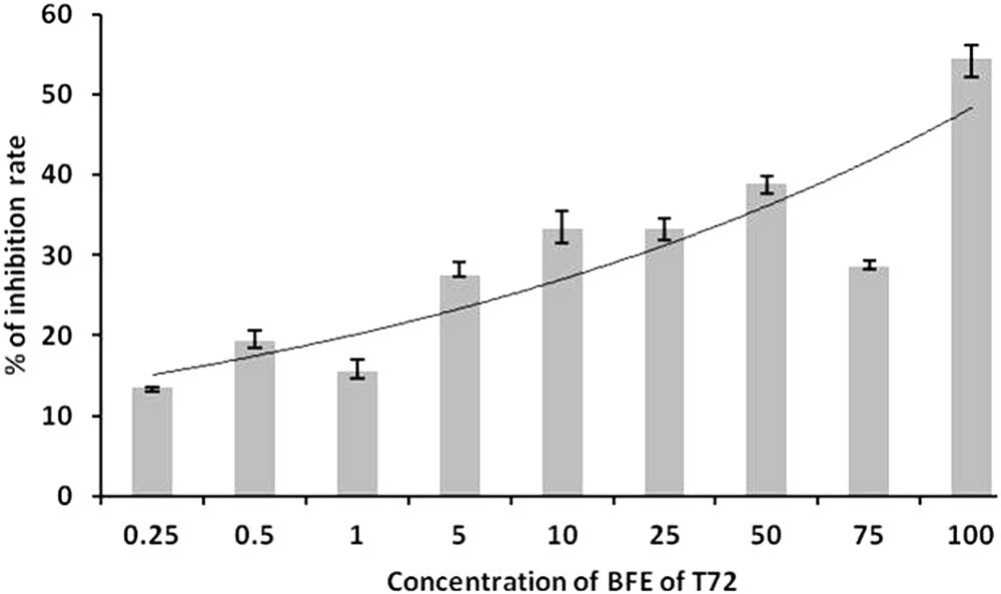
Effect of bioactive fungal metabolite (BFE) derived from *T. harzianum* strain CCTCC-RW0024 (μl/100 ml of PDA) on growth inhibition rate of FG.

This experiment we extracted and partially purified the fungal secondary metabolites (SMs) in *T. harzianum* strain CCTCC-RW0024 by using column chromatography and Gas chromatography. The predominant volatile compounds were as predicted by GC-MS analysis, H-[1]Benzopyrano[3,4-b]pyridin-5-one, 9-amino-1,2,3,4-tetrahydro-, Pyridazine-3,6(1 H,2 H)-dione, 1-(4-fluorophenyl)-, Benzenamine, 4-cyclohexyl-, Dehydroacetic Acid, o-Cyanobenzoic acid, Eicosanoic acid, Pentanedioic acid, 2-oxo-, dimethyl ester, Ethanone, 1-(1H-pyrazol-4-yl)-, 3-Octyne (Supplementary Fig. 2). These compounds were subjected to molecular docking against growth and pathogenicity related to transcriptional cofactor FgSWi6 of FG (Supplementary Table 5). Further, the effect of this bioactive fungal extract (BFE) was tested against the growth of FG in PDA plate assay at different concentrations of 0.25–100 μg/100 ml of PDA and whereas CK1 (FG grown without incorporation of BFE in PDA), CK_2_ (FG grown with the incorporation of methanol alone in PDA). The growth of FG was significantly decreased with increase of BFE in PDA (Fig. 6). The percentage of growth inhibition (Fig. 7) varied significantly between the concentrations of BFE (p < 0.05) and it was found high in 100 μg (54.54%) and low in 0.25 μg (13.51%).

### *In silico* molecular docking analysis

**Figure 8:**
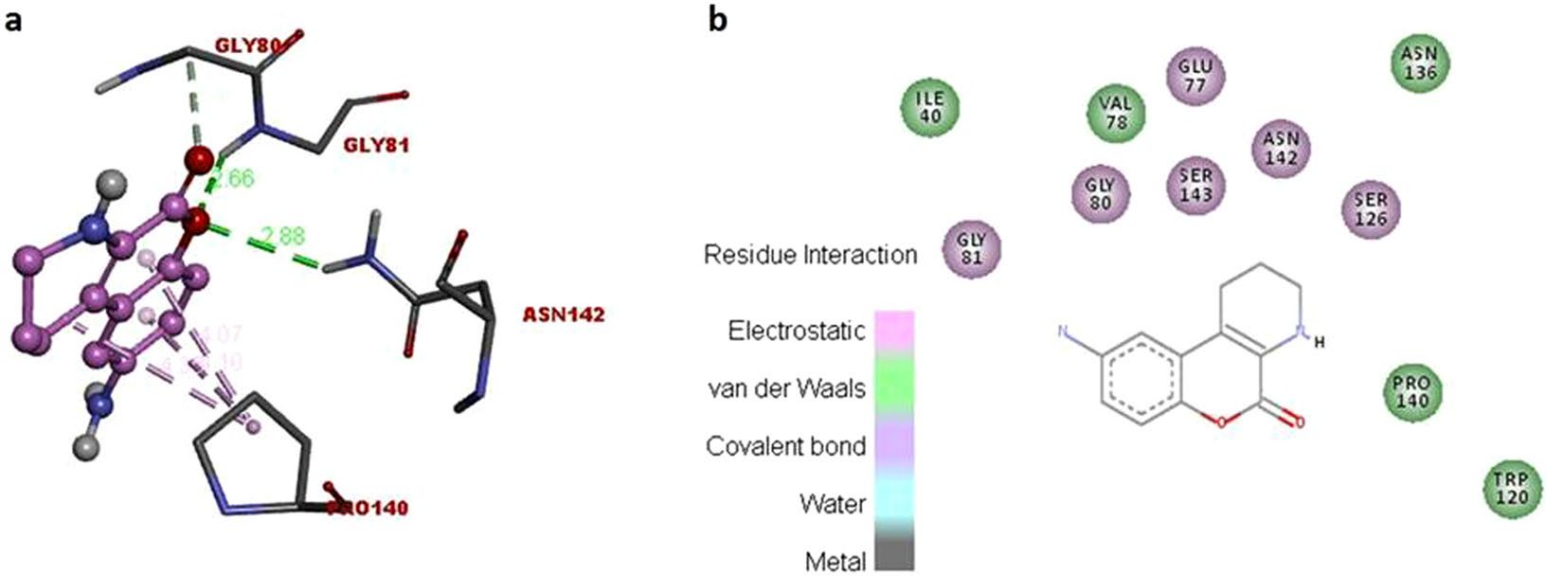
*In silico* molecular docking analysis of partially purified BFE against growth and pathogenicity related transcriptional cofactor FG. (**a**) 3D structure of interactions between pathogenicity related transcriptional cofactor FgSWi6 and partially purified compound H-[1]Benzopyrano[3,4-b]pyridin-5-one, 9-amino-1,2,3,4-tetrahydro-, (**b**) 2D structure of binding site of cofactor FgSWi6 and H-[1]Benzopyrano[3,4-b]pyridin-5-one, 9-amino-1,2,3,4-tetrahydro-.

A total of eight partially identified compounds of *T. harzianum* strain CCTCC-RW0024 were tested against growth and pathogenicity related transcriptional cofactor FgSWi6 of FG by molecular docking method. Among the tested compounds, the H-[1]Benzopyrano[3,4-b]pyridin-5-one, 9-amino-1,2,3,4-tetrahydro- showed high inhibitory energy score of −6.4 (Supplementary Table 5). This potent compound was found to interact with PRO140, TRP120, SER126, ASN142, SER143, GLY80, GLY81, ILE40, VAL78, GLU77, and ASN136 with the hydrogen bond distance of 3.46, 2.66, 2.88, 4.07, 4.10, 4. 35 (Fig. 8a,b). This binding capacity was expected to inhibit the growth and pathogenicity related transcriptional cofactor FgSWi6 of FG. However, this experiment requires further purification and characterization of SMs in *T. harzianum* strain CCTCC-RW0024 by using multiple approaches (LC-MS/MS, NMR, etc).

## Discussion

Maize stalk rot is ubiquitously found across the globe, in which the causal organism *Fusarium graminearum* is able to infect root and basic stem throughout plant growth with typical symptoms at late stage of the growth at grain filling of maize development. So far, there is no good measure to control the soil-borne disease. In recent years, we established *Trichoderma* as potent BCAs to treat maize seeds or rhizosphere soil in farming fields, which has resulted in a significant decrease of disease by 60% or above (data not shown). *Trichoderma* spp., are known to reduce the plant pathogenic attack and also increase the plant growth and yield through the enhancement of growth hormones, soil fertility, and increment of plant beneficial microbiome^27–29^. The biocontrol performance of *Trichoderma* strains in the farming field varies based on environments impact, host-pathogen specificity and stability. Therefore, isolation and screening of *Trichoderma* species against FG from different ecosystems according to the host-pathogen specificity could provide enhanced insight of BCAs. In this study, we screened a total of 100 *Trichoderma* isolates against the FG, and reported a potent strain *T. harzianum* CCTCC-RW0024 with significant antagonistic activity (96.30%), disease reduction (86.66%), biocontrol-related hydrolytic enzyme and gene expression.

Also, we found that this strain significantly increased the maize rhizosphere beneficial microbial community. The pyrosequencing indicated that exogenous inoculation of the strain CCTCC-RW0024 in maize rhizosphere could increase plant growth promoting acidobacteria (18.4%), decreased 66% of FG and increased the plant growth. This strain could also increase the plant beneficial microbiome in maize rhizosphere as evident by qRT-PCR and electrophoresis assay of FG. Similarly, several researchers have reported that acidobacteria act as plant growth promoting bacteria^30, 31^.

Colonization of the *Trichoderma* strains in maize root playsan important role in the function of mycoparasitism process^12^. This was confirmed through qRT-PCR quantification and microscopic observation of eGFP tagged strain *T. harzianum* CTCC -RW0024 in maize root. Moreover qRT-PCR results indicated that the colonization of strain CCTCC-RW0024 significantly reduced the FG in maize root (Fig. 2e). Similarly, *Trichoderma* strains are known to prevent colonization of FG in maize roots^1, 2^. The results also revealed that exogenous inoculation of *T. harzianum* strain CCTCC-RW0024 could significantly increase the plant growth, plant biomass and reduce the pathogen attack.Similar results are also found in the work of Akladious and Abbas^32^.

Antagonistic activity of the *Trichoderma* strains is generally related to the hydrolytic enzyme activity especially chitinase (chitin) and β (1,3) glucanase (pachyman). These enzymes are closely related to mycoparasitism, and on the other hand, they are contributing to the host induced systemic resistance (ISR) to pathogen infection^4, 33, 34^. In our case, CWDEs from *Trichoderma* CCTCC-RW0024 played multiple functions in the biocontrol process against stalk rot^35, 36^ (Supplementary Table 2, Fig. 1). We also found that there was a positive correlation of 19%, and 50% of the antagonistic activity and enzyme activity of chitinase and β (1,3) glucanase respectively (Supplementary Fig. 1). This means that biocontrol activity of *Trichoderma* strain depends on the combined action of multiple enzymes.

*Trichoderma* spp. mediated biological control of plant pathogens is a synergistic action of CWDEs, secondary metabolites, peptaibols^2, 4, 13, 14^. The volatile organic compound in *Trichoderma* spp. has the ability to increase the plant growth^9, 16, 37^, the small protein sm1 and sm2 in *Trichoderma virens* is significantly involved in fungal root interactions^38^. Our work also attempted the extraction and partial purification of bioactive fungal extract (BFE) from *T. harzianum* strain CCTCC-RW0024, analysed the inhibition effect of FG by following a molecular docking technique^19^ to study the inhibitory capacity of strain CCTCC-RW0024 derived molecules on transcriptional cofactor FgSWi6. The results showed that BFE was potent to inhibit the FG in PDA Petri dishes (Fig. 6); Further, the strain CCTCC-RW0024 derived partially identified molecules H-[1]Benzopyrano[3,4-b]pyridin-5-one, 9-amino-1,2,3,4-tetrahydro- showed high inhibitory energy score of −6.4 against transcriptional cofactor FgSWi6 of FG (Supplementary Table 5; Fig. 8). Therefore, further study on the purification, characterization and underlying molecular mechanisms of the compound H-[1]Benzopyrano[3,4-b]pyridin-5-one, 9-amino-1,2,3,4-tetrahydro by using multiple approaches (LC/LC-MS, HPLC or NMR) deserves due attention.

## Conclusion

This work reports *T. harzianum* strain CCTCC-RW0024 as a potent biocontrol agent against *Fusarium graminearum* a causal agent of maize stalk rot. In addition, this work indicated that inoculation of *T. harzianum* strain CCTCC-RW0024 in infected maize field could control stalk rot through different mechanisms or composite mechanisms including mycoparasitism, ISR, the increment of the maize rhizosphere microbiome and soil fertility. This work emphasized to study the genetic, transcriptomes and specific characterization of SMs in *T. harzianum* strain CCTCC-RW0024 responsible for the interaction of maize root. Therefore, further molecular study and SMs characterization will help to understand the molecular mechanisms through which *T. harzianum* acts as biocontrol agents.

## Materials and Methods

### Fungal isolates, Medium, and culture preservation

A total of 100 *Trichoderma* isolates were tested in this study for control of stalk rot fungal pathogen *Fusarium graminearum* (FG). *Trichoderma* isolates used in this were previously isolated from the non-agricultural ecosystem in China^39^. The culture of *F. graminearum* was received from Prof. Dr. Jie Chen, Shanghai Jiao Tong University, P. R. China.

### *In vitro* assay

In this assay, a total of 100 *Trichoderma* isolates were selected to test the antagonistic activity according to the method of Dennis and Webster^40^. In detail, the mycelial disk (5 mm diameter) of 7 days old growing edge of *Trichoderma* and FG were placed on PDA Petri disk (Size- 90 × 15 mm) at an equal distance of opposite direction. The control was maintained without *Trichoderma* disk. The incubation and the method of incubation rate calculation were described in our previous study^19^.

### Biocontrol-related enzymes and gene expressions

Based on the previous *in vitro* antagonistic assay, a total of 10 *Trichoderma* strains *Trichoderma asperellum* CCTCC-SBW0102 (T3), *T. aureoviride* CCTCC-SBW0005 (T9), *T. harzianum* CCTCC-SBW0101 (T11), *T. asperellum* CCTCC-SBW0109 (T12), *T. atroviride* CCTCC-SBW0016 (T21), *T. asperellum* CCTCC-SBW0052 (T43), *T. asperellum* CCTCC-SBW0091 (T44), *T. tawa* (T64), *T. harzianum* CCTCC-RW0024 (T72), and *T. harzianum* CCTCC-SBW0181 (T88) were selected for the preliminary enzyme assay^41–43^. The percentage of the enzyme activity was calculated using the following formula as Hydrolysis capacity (%) = Diameter of clear zone (cm)/Diameter of *Trichoderma* colony (cm).

RNA was isolated from the fungal biomass and treated with DNase I according to manufacturers instructions (RNAprep Pure Kit, TIANGEN Biotech (Beijing) Co., Ltd.). It was then reverse transcribed to cDNA according to manufacturer instructions (PrimeScript^TM^ II 1^st^ Strand cDNA synthesis kit, Takara, Clontech, Japan). cDNAs were diluted 1:10 and analysed for the CWDEs such as chitinase (*nag1*), cellulase (*Thph1* & *Thph2*), protease (*papA*) and glucanase (*exg1*) gene expression by using SuperReal PreMix Plus (SYBR Green; Tiangen Biotech Co. Ltd, China) by an FTC-3000 Real-Time quantitative PCR System (Funglyn Biotech, Canada). The actin gene (act) was used as control (housekeeping gene)^44^. Primer for the Thph1 and Thph2 was designed in this study and other primer sequences were retrieved from Steindorff *et al*.^45^.

### Antagonistic activity in planta

Maize (*Inbred line Huangzao* 4) seeds were surface sterilized according to the method of Morán-Diez *et al*.^46^. The seeds were then allowed to germinate on sterile wet filter paper at 25 °C for 72 h. For the greenhouse experiments, for the *in planta* antagonistic assay, the native soil was collected from local agriculture field. In order to minimize the weeds, nematodes, insects, microbiome in soil, the native soil was subjected to solarization followed by sterilization at 180 °C for 6 h. The greenhouse experiments were carried out in the soil with pH 7.1, and organic matter (0.7%). We selected *T. harzianum* CCTCC-RW0024 for greenhouse experiment based on the screening. Spore suspension of the strain CCTCC-RW0024 was prepared aseptically for greenhouse experiment according to the method of Cappuccino and Sherman^30^. Five ml of CCTCC-RW0024 suspension (3.8 × 10^7^ CFU/ml) was inoculated in the soil. For pathogen inoculation, 1.5 g of FG biomass was inoculated in one kg of soil. The greenhouse experiment was conducted inside the greenhouse with a temperature of 27 ± 2 °C and photosynthetically active radiation of 750 ± 75_mol.m^−2^.s^−1^. Two greenhouse experiments were performed with the objectives (1) to study the *in planta* antagonistic activity. For this experiment, we used sterile soil, and (2) to study the impact of the exogenous application of *T. harzianum* CCTCC-RW0024 on the maize rhizosphere microbiome, for this experiment we used unsterilized native agriculture soil.

A total of four treatments were applied for the greenhouse experiment as follows: CK1-Natural farming soil as such collected from the farming field. For *T. harzianum* strain CCTCC-RW0024 colonization analysis T_1_- Natural farming soil not inoculated with any microbes and amended with 10% vermiculite, T_2_-Natural farming soil inoculated with *T. harzianum* strain CCTCC-RW0024 (T72) and amended with 10% vermiculite, T_3_- Natural farming soil inoculated with FG and amended with 10% Vermiculate, T_4_- Natural farming soil inoculated *T. harzianum* strain CCTCC-RW0024 (T72) and FG and amended with 10% Vermiculate. The pre-germinated seedlings were planted in the pot after a week of microbial inoculation. One month later, shoot length, root length, shoot biomass, and root biomass were measured in the maize seedlings. *Fusarium* stalk rot disease reduction (%) in maize by *T. harzianum* CCTCC-RW0024 treatments was evaluated at an adult stage after the three months of treatment. Diseases reduction was assessed using the formula described by Saravanakumar *et al*.^19^. These experiments were conducted as three replicate per treatments in a randomized trial to study the effect of each treatment on growth of maize.

### RT-qPCR detection of *T. harzianum* and FG colonization

In order to determine the fungal colonization in the maize root, the ITS copies of *T. harzianum* CCTCC-RW0024 and FG were determined using RT-qPCR by designing specific primers for *T. harzianum* CCTCC-RW0024: ITS spacer TH-F (5′-GCATTTCGCTGCGTTCTTCA-3′), ITS spacer TH-R (5′-TAATCTGAGCCTTCTCGGCG-3′), FG: ITS spacer FG-F (5′-GAGGGTTGAAATGACGCTCG-3′) and ITS spacer FG-R (5′-ACGGATCTCTTGGTTCTGGC-3′) colonization was determined in all the four treatments.

For the RT-qPCR assay, three plant roots were randomly collected from each treatment. Maize roots samples were prepared according to the methods described by Saravanakumar *et al*.^19^. The root DNA was extracted from 100 mg of fresh maize root by using a DNA extraction kit (Plant Genomic DNA Kit, TIANGEN Biotech (Beijing) Co., Ltd.). The number of internal transcribed spacer (ITS) copies of *Trichoderma* and FG were determined by using Super Real PreMix Plus (SYBR Green, TIANGEN Biotech (Beijing) Co., Ltd.) by an FTC-3000 Real-Time quantitative PCR System (Funglyn Biotech, Canada).

### Generation of eGFP-Tagged *T. harzianum*

The eGFP expressing cassette sequence was amplified from the plasmid pCPXHY1eGFP with the primers P*gpd*-F/T*gpd*-R^47^. The eGFP expression of *T. harzianum* CCTCC-RW0024 was generated by ATMT method using the modified plasmid pCAMBIA1300. Resistant colonies were observed on PDA plates supplemented with 200 µg/mL hygromycin. The presence of green fluorescence and the insertion of eGFP expression cassette were confirmed by fluorescent microscopic observation of *T. harzianum* CCTCC-RW0024.

### Analysis of microbiomes by pyrosequencing

In order to analyse the microbial populations the unsterilized native soil were used, a total of four soil, four maize root and one natural farming soil samples such as CK-unsterilized natural farming field soil, T1S- uninoculated soil, T2S-Soil inoculated with *T. harzianum* strain CCTCC-RW0024, T3S-Soli inoculated with FG, T4S-Soil inoculated with *T. harzianum* strain CCTCC-RW0024 and FG, T1R Maize root samples collected from uninoculated soil pot, T2R maize root collected from soil inoculated with *T. harzianum* strain CCTCC-RW0024, T3R- Maize root samples collected from inoculated with FG pot, T4R- Maize root samples collected from inoculated with *T. harzianum* strain CCTCC-RW0024 and FG were subjected to the pyrosequencing experiments. Genomic DNA was extracted from the soil, maize root samples using the TIANamp sediment and plant DNA kit according to manufacturer’s instructions (Tiangen, China). After extraction, the purity of the DNA was tested using a UV spectrophotometer followed by 0.8% agarose gel electrophoresis at a voltage of 120 V for 20 min. The internal transcribed spacer (ITS) regions were amplified for fungal ITS1F (5′-CTTGGTCATTTAGAGGAAGTAA-3′) and 5.8 sRNA gene A2R (5′-CTGCGTTCTTCATCGAT-3′) and bacterial 16S rRNA gene: F343 (5′-TACGGRAGGCAGCAG-3′) and R803 (5′-CTACCAGGGTATCTAATCC-3′)^48^. The PCR reaction was conducted in 20 ng/µl of reaction mixture which consisted of 8.75 µl of ultra pure H_2_O, 5 µl of 5x Q5 Buffer, 5 µl of 5x GC Enhancer, 2 µl of dNTP (2.5 mM), 2 µl of template DNA (2 ng/ µl), 1 µl each forward and reverse primer (10 µM), 0.25 µl of Q5 DNA polymerase. The PCR cycling conditions were set as 4 min at 98 °C, 27 cycles (98 °C for the 30 s, 47.6 °C for 45 s, 72 °C for 1 min), then the final extension at 72 °C for 5 min, and finally the experiment was halted at 10 °C. The PCR products were purified using AMpure Beads followed by the PicoGreen dsDNA assay kit used for the quantification of DNA. Finally, the mixture was pyrosequenced by using Roche 454 GS FLX (Shanghai Personalbio Co., Ltd., China). The sequence data was analyzed by using Qiime (version 1.7.0, http://qiime.org/) followed by the mother (version 1.31.2, http://www.mothur.org/) and further the data was analyzed according to Sun *et al*.^49^. In brief, the below sequences score of 25 and 200 bp length were trimmed and binned into operational taxonomic units (OTUs) using a 97% of the threshold for the bioinformatics and subsequent analysis^49^.

### Extraction of secondary metabolites

*T. harzianum* CCTCC-RW0024 was subjected to extraction of fungal metabolites according to the methods described by Saravanakumar *et al*.^19^. In brief, the culture of CCTCC-RW0024 (3.5 × 10^7^ spores/ml) was inoculated into 20 liters of a production medium with the pH 7.2 and incubated for 31 days at 28 °C at 180 rpm of shaking. After the incubation, the microbial culture was subjected to ethyl acetate extraction and the crude extract was concentrated using a rotary evaporator. Finally, the concentrated extracts were dissolved in 90% methanol for further partial purification. A total of 10 fractions were collected from the column chromatography and subjected to antifungal activity assay against plant pathogen FG at different concentrations (1–100 µl. 250 ml^−1^ of PDA medium). The selected active fractions were partially studied by the gas chromatography-mass spectrometer (GC-MS; AutoSystem XL GC/TurboMass MS).

### Preparation of transcriptional cofactor FgSWi6

In order to study the inhibitory effect of *Trichoderma* metabolites on pathogenicity related transcriptional cofactor FgSWi6 of FG (Liu *et al*.^24^). An *in silico* molecular docking method was applied^19^. The FASTA format of an amino acid sequence of FgSWi6 (sequence length of 807 residues and molecular weight of 87663.2) was retrieved from protein database of National centre for Biotechnology information (NCBI). We used the ClustalW and MODELLER v9.12 for the sequence alignment for FgSWi6 with the template protein. The 3D structure FgSWi6 was predicted using template amino acids sequence by Swiss-Model (https://swissmodel.expasy.org/). Of the three models, the one with the lowest score was taken for energy minimization using the CHARMm force field in Discovery Studio v4.1. The modeled 3D structure of FgSWi6 was used for molecular docking studies.

### Molecular docking analysis

Fungal metabolites were partially identified based on GC-MS analysis.Further the compound structures were predicted by using the ChemSketch software (http://www.acdlabs.comfrom) with the assistance of NCBI-PubChem database (http://www.ncbi.nlm.nih.gov/pccompound). The predicted structure of compound molecule was saved as MOL file for docking study^19^. The interaction between the *Trichoderma*-derived metabolites and target transcriptional cofactor FgSWi6 was studied by using ArgusLab 4.0.1 and Discover Studio Version 4.0 (Accelry’s Software Inc. the USA). The mechanism of ligand placement was based on binding site position, therefore, the fitting points were added to hydrogen bonding groups on the target protein and *Trichoderma*-derived molecule (ligand). Scoring function was implemented in docking programme by making various assumptions and implications in the evaluation of model complexes, which includes terms of hydrogen bonds employed by Discovery Studio to rank the docked bases and to assess the binding site and the number of present rotatable bonds.

### Statistical analysis

All experiments were repeated three times. The significance between variables among *Trichoderma* species in enzymes experiments and treatments of greenhouse experiments, a suite of statistical analysis (SPSS 11.5) and MS Excel-2007 was used to find the mean and standard error. ANOVA (one-way classifications) with Duncan Post hoc multiple comparisons was applied. The heat map for the mycoparasitism related genes was drawn by using HemI: A Toolkit for Illustrating Heatmaps^50^.

## Electronic supplementary material

Supporting information
