# Supplementary material for: Effect of Trichoderma harzianum on maize rhizosphere microbiome and biocontrol of Fusarium Stalk rot
Source: Sci Rep. 2017 May 11;7:1771. doi: 10.1038/s41598-017-01680-w (PMC5431858; doi:10.1038/s41598-017-01680-w)
Supplement: Supplementary file 1 — Supporting information [file 41598_2017_1680_MOESM1_ESM.doc]

**Effect of *Trichoderma harzianum* on maize rhizosphere microbiome and biocontrol of *Fusarium* Stalk rot**

Kandasamy Saravanakumar^1,2,3¶^, Yaqian Li^1,2,3¶*^, Chuanjin Yu^1,2,3^, Qiang-qiang Wang^1,2,3^, Meng Wang^1,2,3^, Jianan Sun^1,2,3^, Jin-xin Gao^1,2,3^, Jie Chen^1,2,3^*

^1^School of Agriculture and Biology, Shanghai Jiao Tong University, Shanghai, P.R. China; ^2^State Key Laboratory of Microbial Metabolism, Shanghai Jiao Tong University, Shanghai, P. R. China;

^3^Key Laboratory of Urban Agriculture (South), Ministry of Agriculture, Shanghai, P.R. China

**Competing Interests:** The authors have declared that no competing interests exist

^¶^ Shared first authorship

***Corresponding author:**

Prof. Dr. Jie Chen

Shanghai Jiao Tong University
Department of Environment and Resource, School of Agriculture and Biology
800 Dongchuan Rd
Cuddalore (Dt)
Shanghai, Shanghai 200240
China

Tel: +8621-34206141; Fax: +8621-34206141 E. mail: [jiechen59@sjtu.edu.cn](mailto:jiechen59@sjtu.edu.cn)

*Co corresponding author

Dr. Yaqian Li

Shanghai Jiao Tong University
Department of Environment and Resource, School of Agriculture and Biology
800 Dongchuan Rd, Shanghai, Shanghai 200240， P.R.China

Tel & Fax: 021-34206620; E. mail: [lauren@sjtu.edu.cn](mailto:lauren@sjtu.edu.cn)

**Supplementary table. 1. *Invitro* antagonist activity of *Trichoderma* isolates against *Fusarium graminearum* (The mean percentage of inhibition of pathogen in dual culture, degrees of freedom-3). The top ten highly potential isolates are highlighted as** BOLD

| Isolates No | CCTCC culture collection code | Isolate code | Organism | % |
| --- | --- | --- | --- | --- |
| 1 | CCTCC-SBW0004 | BHMT4 | *Trichoderma aureoviride* | 93.14 |
| 2 | CCTCC-SBW0007 | CHI1 | *T. asperellum* | 73.53 |
| **3** | **CCTCC-SBW0102** | **ZHMT4** | ***T. asperellum*** | **92.30** |
| 4 | CCTCC-SBW0114 | ZHYAQT2 | *T. harzianum* | 58.82 |
| 5 | CCTCC-SBW0108 | ZHMT9 | *T. harzianum* | 65.69 |
| 6 | CCTCC-SBW0106 | ZHMT7 | *T. asperellum* | 54.90 |
| 7 | CCTCC-SBW0096 | ZHMT13 | *T. harzianum* | 74.45 |
| 8 | CCTCC-SBW0098 | ZHMT2 | *T. harzianum* | 71.37 |
| **9** | **CCTCC-SBW0005** | **BJ1** | ***T. aureoviride*** | **85.29** |
| 10 | CCTCC-SBW0095 | ZHMT12 | *T. harzianum* | 60.78 |
| 11 | **CCTCC-SBW0101** | **ZHMT3** | ***T. harzianum*** | **89.22** |
| 12 | **CCTCC-SBW0109** | **ZHMT9** | ***T. asperellum*** | **93.14** |
| 13 | CCTCC-SBW0103 | ZHMT5 | *T. harzianum* | 62.75 |
| 14 | CCTCC-SBW0105 | ZHMT6 | *T.asperellum* | 58.82 |
| 15 | CCTCC-SBW0094 | ZHMT1 | *T.harzianum* | 60.78 |
| 16 | CCTCC-RW0001 | CHIPU1 | *T.harzianum* | 62.75 |
| 17 | CCTCC-SBW0097 | ZHMT14 | *T. harzianum* | 63.73 |
| 18 | CCTCC-SBW0015 | CHI5 | *T. asperellum* | 69.61 |
| 19 | CCTCC-SBW0022 | CHIPUFI2 | *T. atroviride* | 60.78 |
| 20 | CCTCC-SBW0021 | CHIa | *T. atroviride* | 73.45 |
| **21** | **CCTCC-SBW0013** | **CHI4** | ***T. asperellum*** | **77.45** |
| 22 | CCTCC-SBW0016 | CHI5 | *T. atroviride* | 67.65 |
| 23 | CCTCC-SBW0006 | CHI (WIN) | *T. harzianum* | 70.59 |
| 24 | CCTCC-SBW0019 | CHI8 | *T. asperellum* | 65.69 |
| 25 | CCTCC-SBW0018 | CHI7 | *T. atroviride* | 62.75 |
| 26 | CCTCC-SBW0009 | CHI12 | *T. atroviride* | 73.53 |
| 27 | CCTCC-SBW0020 | CHI9 | *T. asperellum* | 71.37 |
| 28 | CCTCC-SBW0023 | PU2 | *T. longibrachiatum* | 70.59 |
| 29 | CCTCC-SBW0008 | CHI11 | *T. atroviride* | 65.69 |
| 30 | CCTCC-SBW0025 | PUFP3 | *T. atroviride* | 61.76 |
| 31 | CCTCC-SBW0017 | CHI6 | *T. asperellum* | 69.41 |
| 32 | CCTCC-SBW0011 | CHI2 | *T. asperellum* | 65.69 |
| 33 | CCTCC-SBW0024 | PUFP1 | *T. atroviride* | 65.69 |
| 34 | CCTCC-SBW0104 | ZHMT5 | *T. asperellum* | 57.84 |
| 35 | CCTCC-SBW0014 | CHI4 | *T.asperellum* | 66.67 |
| 36 | CCTCC-SBW0012 | CHI3 | *T. asperellum* | 71.57 |
| 37 | CCTCC-SBW0099 | ZHMT20 | *T. harzianum* | 61.76 |
| 38 | CCTCC-SBW0107 | ZHMT8 | *T. asperellum* | 62.75 |
| 39 | CCTCC-SBW0100 | ZHMT21 | *T. harzianum* | 60.78 |
| 40 | CCTCC-SBW0010 | CHI15 | *[T.asperellum](http://blast.ncbi.nlm.nih.gov/Blast.cgi#alnHdr_363399352)* | 59.80 |
| 41 | CCTCC-SBW0050 | FJWT1 | *T.harzianum* | 72.35 |
| 42 | CCTCC-SBW0054 | FJWT4 | *T. asperellum* | 72.55 |
| **43** | **CCTCC-SBW0052** | **FJWT2** | ***T. asperellum*** | **92.16** |
| **44** | **CCTCC-SBW0091** | **SYXC1** | ***T.asperellum*** | **82.50** |
| 45 | CCTCC-SBW0092 | SYXC3 | *T. atroviride* | 67.45 |
| 46 | CCTCC-SBW0057 | FJWT7 | *T. asperellum* | 59.80 |
| 47 | CCTCC-SBW0055 | FJWT5 | *T. harzianum* | 69.45 |
| 48 | CCTCC-SBW0090 | STWT5 | *T. koningiopsis* | 74.51 |
| 49 | CCTCC-SBW0058 | FJWT8 | *T. asperellum* | 70.39 |
| 50 | CCTCC-SBW0093 | SYXR1 | *T. harzianum* | 67.65 |
| 51 | CCTCC-SBW0056 | FJWT6 | *T. atroviride* | 53.92 |
| 52 | CCTCC-SBW0053 | FJWT3 | *T. asperellum* | 65.69 |
| 53 | CCTCC-SBW0089 | SYC2 | *T. atroviride* | 58.82 |
| 54 | CCTCC-SBW0076 | STWT1 | *T. longibrachiatum* | 63.73 |
| 55 | CCTCC-SBW0080 | STWT4 | *T. asperellum* | 70.59 |
| 56 | CCTCC-SBW0088 | SW2 | *T. velutinum* | 75.49 |
| 57 | CCTCC-SBW0084 | STWT6 | *T. asperellum* | 73.33 |
| 58 | CCTCC-RW0021 | ZWPUEB12 | *T. harzianum* | 38.24 |
| 59 | CCTCC-RW0027 | ZWPUEB3 | *T. asperellum* | 74.45 |
| 60 | CCTCC-RW0002 | ZQTR1 | *T. longibrachiatum* | 73.33 |
| 61 | CCTCC-RW0008 | ZWPBG3 | *T. atroviride* | 66.67 |
| 62 | CCTCC-RW0018 | ZWPUEB | *T. harzianum* | 72.35 |
| 63 | CCTCC-RW0020 | ZWPUEB11 | *T. harzianum* | 74.31 |
| **64** | **CCTCC-RW0023** | **ZWPUEB14** | ***T. tawa*** | **90.20** |
| 65 | CCTCC-RW0022 | ZWPUEB13 | *T. harzianum* | 67.45 |
| 66 | CCTCC-RW0012 | ZWPBG7 | *T. asperellum* | 73.33 |
| 67 | CCTCC-RW0019 | ZWPUEB10 | *T. harzianum* | 64.31 |
| 68 | CCTCC-RW0017 | ZWPH1 | *T. viridescens* | 71.37 |
| 69 | CCTCC-RW0011 | ZWPBG6 | *T. asperellum* | 63.33 |
| 70 | CCTCC-RW0004 | ZWPBG1 | *T. asperellum* | 66.08 |
| 71 | CCTCC-RW0009 | ZWPBG4 | *T. koningiopsis* | 70.59 |
| **72** | **CCTCC-RW0024** | **ZWPUEB15** | ***T. harzianum*** | **96.30** |
| 73 | CCTCC-RW0010 | ZWPBG5 | *T. koningii* | 75.49 |
| 74 | CCTCC-RW0006 | ZWPBG10 | *T. harzianum* | 83.33 |
| 75 | CCTCC-RW0013 | ZWPBG8 | *T. asperellum* | 38.24 |
| 76 | CCTCC-RW0015 | ZWPBG9 | *T. harzianum* | 77.45 |
| 77 | CCTCC-RW0003 | ZEPUEB7 | *T. aureoviride* | 63.33 |
| 78 | CCTCC-RW0007 | ZWPBG2 | *T. asperellum* | 66.67 |
| 79 | CCTCC-SBW0051 | FJWT14 | *T. viride* | 72.35 |
| 80 | CCTCC-RW0028 | ZWPUEB4 | *T.asperellum* | 74.31 |
| 81 | CCTCC-RW0025 | ZWPUEB2 | *T.longibrachiatum* | 72.35 |
| 82 | CCTCC-RW0005 | ZWPBG1 | *T.asperellum* | 73.45 |
| 83 | CCTCC-RW0014 | ZWPBG8 | *T. asperellum* | 63.33 |
| 84 | CCTCC-RW0016 | ZWPBG9 | *T. virens* | 74.31 |
| 85 | CCTCC-RW0026 | ZWPUEB2 | *T. atroviride* | 71.37 |
| 86 | CCTCC-SBW0184 | ZNE8 | *T. harzianum* | 73.33 |
| 87 | CCTCC-SBW0185 | ZNE9 | *T. harzianum* | 66.08 |
| **88** | **CCTCC-SBW0181** | **ZNE5** | ***T. harzianum*** | **82.35** |
| 89 | CCTCC-SBW0179 | ZNE1 | *T. harzianum* | 70.59 |
| 90 | CCTCC-SBW0156 | ZNBW12 | *Trichoderma harzianum* | 75.49 |
| 91 | CCTCC-SBW0154 | ZNBW10 | *T. harzianum* | 73.33 |
| 92 | CCTCC-SBW0178 | ZNCFW8 | *T. atroviride* | 38.24 |
| 93 | CCTCC-SBW0129 | ZNAF15 | *T. atroviride* | 74.45 |
| 94 | CCTCC-SBW0169 | ZNCF17 | *T. harzianum* | 73.33 |
| 95 | CCTCC-SBW0177 | ZNCFW7 | *T. atroviride* | 66.67 |
| 96 | CCTCC-SBW0170 | ZNCF18 | *T. atroviride* | 72.35 |
| 97 | CCTCC-SBW0155 | ZNBW11 | *T. harzianum* | 74.31 |
| 98 | CCTCC-SBW0158 | ZNBW15 | *T. harzianum* | 72.35 |
| 99 | CCTCC-SBW0205 | ZNR19 | *T. atroviride* | 67.45 |
| 100 | CCTCC-SBW0208 | ZNR4 | *T. harzianum* | 73.33 |

*Supplementary* *Table.2. Hydrolytic capacity of potent selected Trichoderma isolates by microbial plate assay. The results showed mean ± standard error (Degrees of freedom-3). One way ANOVA followed by multiple comparison of Duncan test.*

| Isolates  no | CCTCC culture collection code | Hydrolytic capacity (%) | | | |
| --- | --- | --- | --- | --- | --- |
|  |  | Chitin | Gelatin | CMC | pachyman |
| T3 | CCTCC-SBW0102 | 78.3±2.36^d^ | 40.5±8.65^a^ | 51.7±2.56^b^ | 76.2±7.25^c^ |
| T9 | CCTCC-SBW0005 | 6.3±1.56^a^ | 41.4±2.56^a^ | 34.5±1.45^a^ | 35.7±3.45^a^ |
| T11 | CCTCC-SBW0101 | 33.3±4.58^b^ | 41.7±2.54^a^ | 0 | 71.4±1.58^c^ |
| T12 | CCTCC-SBW0109 | 34.3±2.65 | 84±1.54^b^ | 72.4±4.56^c^ | 78.6±6.54^c^ |
| T21 | CCTCC-SBW0013 | 21.7±3.25^b^ | 75±4.25^b^ | 3.4±5.15^a^ | 76.2±2.56^c^ |
| T43 | CCTCC-SBW0052 | 50±1.25^c^ | 88.9±2.56b | 20.7±2.64^a^ | 50±3.65^b^ |
| T44 | CCTCC-SBW0091 | 50±4.56^c^ | 46.2±4.51^a^ | 51.7±1.56^b^ | 76.2±1.45^c^ |
| T64 | CCTCC-RW0023 | 62.5±2.36^c^ | 84.2±3.65^b^ | **89.7±4.56^c^*** | 28.6±2.52^a^ |
| **T72** | CCTCC-RW0024 | **85.7±1.56^d^*** | **88.0±1.58^b^*** | 79.3±2.54^c^ | **83.3±2.54^d^*** |
| T88 | CCTCC-SBW0181 | 75.9±2.65^d^ | 71.9±4.56^b^ | 75.9±3.26^c^ | 52.4±3.65^b^ |

*Highest hydrolytic capacity (%), CMC-Carboxymethyl cellulose

Supplementary Table.3. Taxonomical distribution of bacterial OTUs

Supplementary Table.4. Taxonomical distribution of bacterial OTUs

**Supplementary Table.5. Molecular docking study against growth and pathogenicity related transcriptional cofactor FgSWi6 of FG**

| **S.No.** | **Pubchem Id** | **Compound Name** | **Mol.Wt** | **Mol. Formula** | **Docking Score** |
| --- | --- | --- | --- | --- | --- |
| 1 | 167621435 | H-[1]Benzopyrano[3,4-b]pyridin-5-one, 9-amino-1,2,3,4-tetrahydro- | C_12_ H_12_ N_2_ O_2_ | 216.236 | **-6.4** |
| 2 | 609743 | Pyridazine-3,6(1H,2H)-dione, 1-(4-fluorophenyl)- | C_10_ H_7_ F N_2_ O_2_ | 206.173 | -5.9 |
| 3 | 80764 | Benzenamine, 4-cyclohexyl- | C_12_ H_17_ N | 175.27 | -5.8 |
| 4 | 122903 | Dehydroacetic Acid | C_8_ H_8_ O_4_ | 168.147 | -5.4 |
| 5 | 138061 | o-Cyanobenzoic acid | C_8_ H_5_ N O_2_ | 147.131 | -5.1 |
| 6 | 10467 | Eicosanoic acid | C_20_ H_40_ O_2_ | 312.53 | -5 |
| 7 | 24013 | Pentanedioic acid, 2-oxo-, dimethyl ester | C_7_ H_10_ O_5_ | 174.157 | -4.5 |
| 8 | 491655 | Ethanone, 1-(1H-pyrazol-4-yl)- | C_5_ H_6_ N_2_ O | 110.114 | -4.3 |


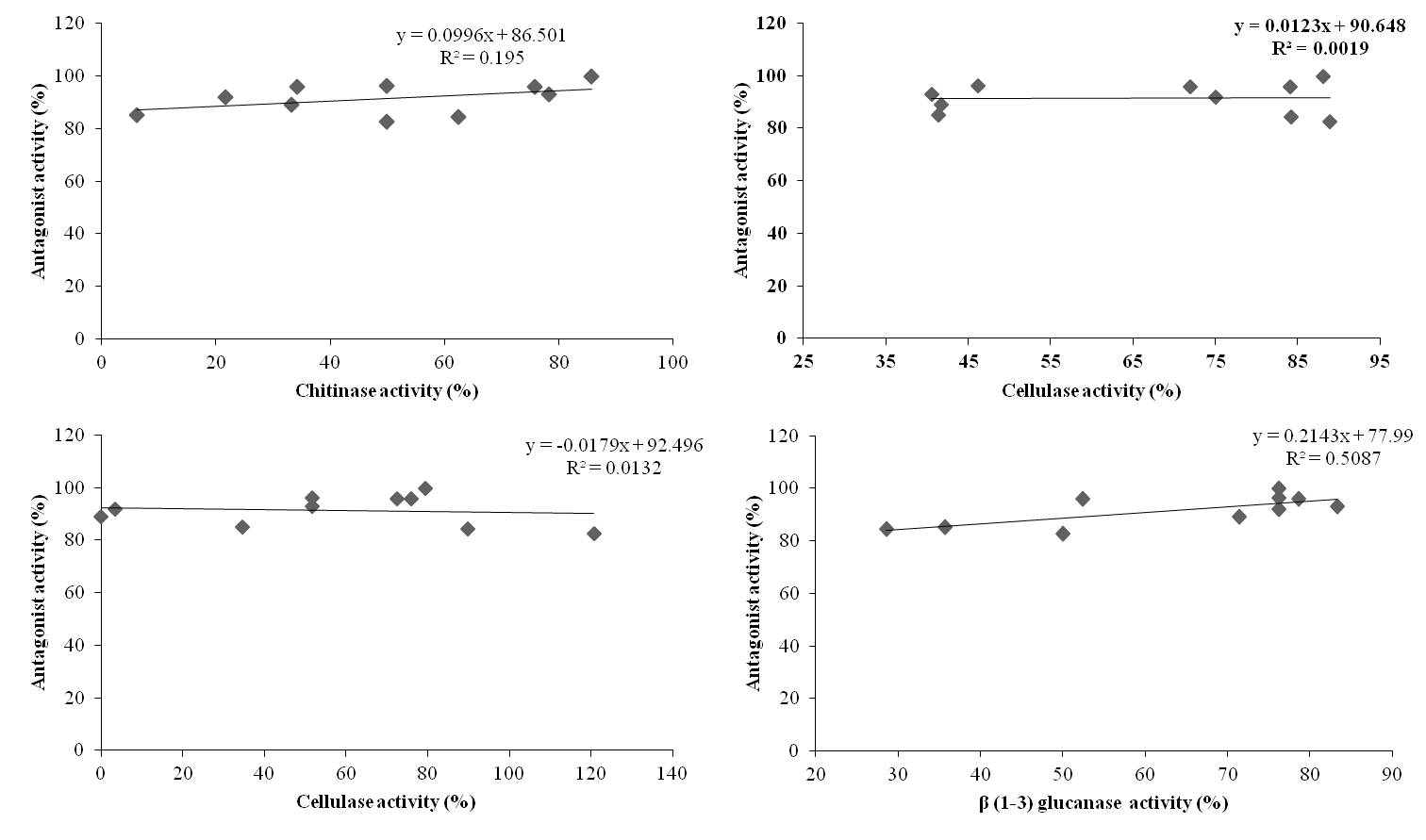


Supplementary Fig. 1. Relation between CWDEs activity and antagonist activity of *Trichoderma*

Supplementary Fig.2. GC-MS chromatogarph of BFE derived from *T. harzianum* strain CCTCC-RW0024.
